# Effects of Genetic Origin of Honeybees and Climate on Prevalence and Infestation Levels of Varroa

**DOI:** 10.3390/ani13203277

**Published:** 2023-10-20

**Authors:** Claudia García-Figueroa, Francisco Javier Ramírez-Ramírez, Laura Yavarik Alvarado-Avila, Miguel Enrique Arechavaleta-Velasco

**Affiliations:** Centro Nacional de Investigación Disciplinaria en Fisiología y Mejoramiento Animal, Instituto Nacional de Investigaciones Forestales, Agrícolas y Pecuarias, Querétaro 76280, Mexico; garcia.claudia@inifap.gob.mx (C.G.-F.); ramirez.javier@inifap.gob.mx (F.J.R.-R.); alvarado.laura@inifap.gob.mx (L.Y.A.-A.)

**Keywords:** honeybees, Varroa, morphotype, haplotype, climate type, prevalence, infestation levels

## Abstract

**Simple Summary:**

Varroa is a parasite of honeybees and is one of the main problems that beekeeping faces worldwide: it affects honey production and is involved in colony losses reported in some parts of the world. The objective of this study was to assess the effect of honeybee genetic origin, climate type and the interactions between these two variables on the prevalence and infestation levels of Varroa in a large population of 1134 colonies. The morphotype, haplotype and climate type of each colony were determined. The results indicate that the climate has an effect on the prevalence and infestation levels of Varroa: both were higher in the temperate sub-humid climate than in the semi-warm climate and the warm sub-humid climate. The morphotype has no effect: there were no differences between Africanized, European and Hybrid honeybees for the prevalence and infestations levels of Varroa. The haplotype has an effect on prevalence but not on infestation levels, and the African haplotype has a higher prevalence than the European haplotype, but no differences were found between the two haplotypes for infestation levels of Varroa. Correlations between Varroa infestation levels and mean annual temperature, mean annual precipitation, winter precipitation and the Lang index were found.

**Abstract:**

The objective of this study was to evaluate the effect of honeybee genetic origin, climate type and the interactions between these variables on the prevalence and infestation levels of Varroa in a large population of honeybee colonies (n = 1134). For each colony, the morphotype, haplotype and climate type were determined. No differences between the Africanized, European and Hybrid morphotypes were found for the prevalence and infestation levels of Varroa (*p* > 0.05). Differences between honeybee haplotypes were found for the prevalence of Varroa (*p* < 0.05), and the prevalence was higher in the African haplotype than in the European haplotype. No differences between honeybee haplotypes were found for the infestation levels of Varroa (*p* > 0.05). Differences were found between climate type for the prevalence and infestation levels of Varroa (*p* < 0.05): the temperate sub-humid climate had a higher prevalence and higher infestation levels than the semi-warm climate and the warm sub-humid climate. Correlations between the infestation levels of Varroa and mean annual temperature, mean annual precipitation, winter precipitation and Lang index were found.

## 1. Introduction

*Varroa destructor* is a honeybee parasite that affects adult bees and brood. The mite feeds on the fat body tissue of larvae, pupae and adult bees [1] and reproduces on honeybee brood, affecting the normal development of larvae and pupae [2,3,4,5,6]. While varroa is a vector for some viruses that affect honeybees [7,8,9,10,11,12], it is also an important factor for the development of other diseases [11,13,14,15] and it is known that the mite suppresses the immune response of honeybees [16,17,18,19,20].

Varroa is one of the main problems that beekeeping faces worldwide because it affects honey production [17,18,19,21,22,23] and is involved in colony losses reported in some parts of the world [20,21,22,23,24,25,26,27,28,29].

Some studies indicate that Africanized honeybees are less susceptible to Varroa infestation than European honeybees [30,31,32,33,34,35,36,37,38,39]. However, another study indicates that there are no differences in susceptibility and Varroa infestation levels between Africanized and European colonies [38].

Some studies indicate that the genetic origin of the honeybees and the climate type have an effect on the prevalence of the disease and on the mite infestation levels in the colonies [37,40,41].

One of these studies found that both the genetic origin and the climate had an effect on infestation levels. European honeybees had higher infestation levels than Africanized honeybees, and colonies located in cold and temperate climates had higher Varroa infestation levels than colonies located in warm climates [37].

Another of the studies that was conducted in Mexico also found that the genetic origin of the colonies and the climate affected the prevalence of the disease and the infestation levels of the mite in the colonies. European honeybees had a higher prevalence than Africanized honeybees and, in one of the climates included in the study, European honeybees had higher infestation levels than Africanized honeybees, whereas in the other two climates there were no differences between the two genetic groups. In this study, colonies in the temperate sub-humid and subtropical climates were found to have higher infestation levels and a higher prevalence than colonies located in the temperate dry climate [40].

Another study, also conducted in Mexico, found that the genetic origin of the honeybees had an effect on the prevalence and infestation levels of Varroa, but in this study, the climate type had no effect on the two variables. European honeybees had a higher prevalence and higher infestation levels than Africanized honeybees, and, in this study, there were no differences in the prevalence or Varroa infestation levels of colonies located in subtropical and temperate climates [41].

It is important to understand how the genetic origin of honeybees and the climate affect the prevalence of *Varroa destructor* and the infestation levels that the mite can reach in the colonies. The objective of this study was to evaluate the effect of the genetic origin of honeybees, the climate type and the interactions between these two variables on the prevalence and the infestation levels of Varroa in a large population of honeybee colonies.

## 2. Materials and Methods

**Figure 1:**
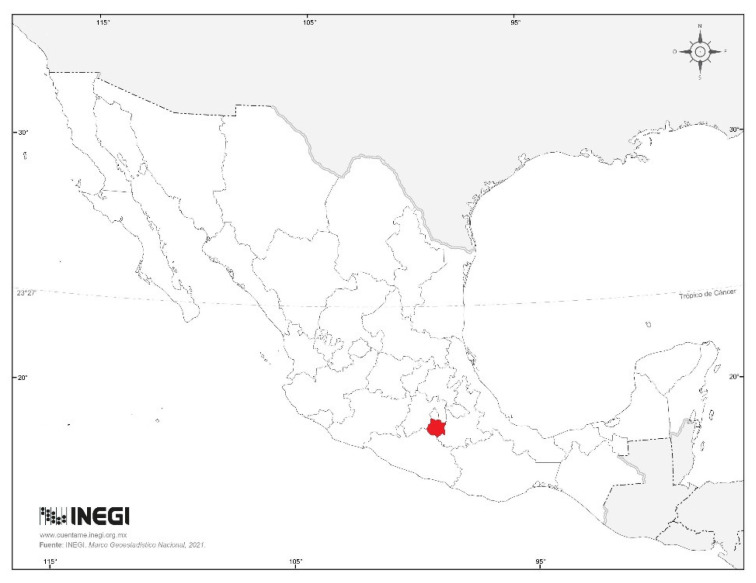
Location of the State of Morelos. Map created with a template available at INEGI website.

The study was conducted in the State of Morelos, located in the central portion of Mexico, in the high plateau beekeeping region (Figure 1). Beekeeping is an important economic activity in Morelos, carried out by more than 700 beekeepers with bees from different genetic origins [42] and in regions with different climate types [43].

### 2.1. Selection of Colonies

The colony inclusion/exclusion restrictions followed in the study were: (1) the colony must not have been treated against Varroa for a period of 12 months; (2) the colony must have a queen; and (3) the colony must have at least three frames with brood, four frames covered with adult bees and two frames of food.

**Figure 2:**
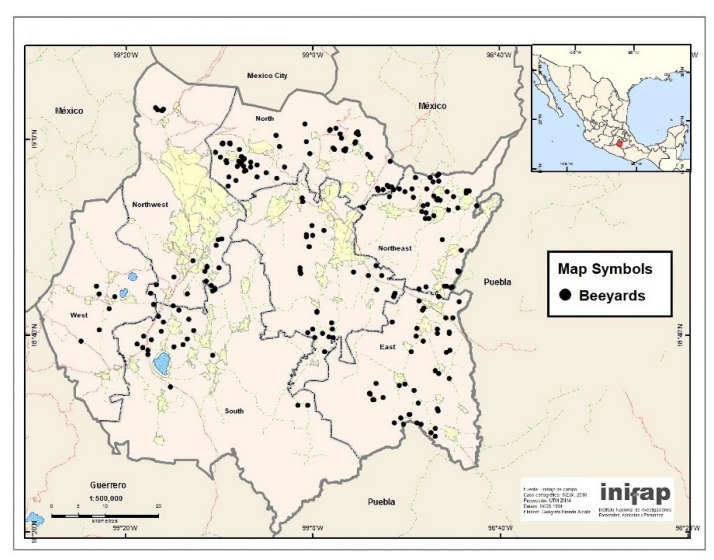
Location of the beeyards included in the study. Map created in ArcGIS 10.5 using a template available at INEGI website.

A total of 1134 honeybee colonies were included in the study. The colonies belonged to 96 beekeepers and were located in 214 beeyards (Figure 2). Two samples of approximately 100 adult bees were collected from each colony from the central frames of the brood chamber in jars with 70% ethanol.

### 2.2. Estimation of Prevalence and Infestation Levels of Varroa Destructor

To determine the number of infested colonies and to estimate the Varroa infestation level of each colony, the two samples were analyzed and the average for each colony was calculated.

To separate the mites from the bees in each sample, the jars were agitated for 15 min with a laboratory mechanical shaker and the content of each jar was poured into a white container where the mites and workers were separated and counted [40,41].

The prevalence was calculated by dividing the number of positive colonies by the total number of colonies. The infestation level of the colonies was determined by the mite infestation in the adult bees, which was calculated by the proportion of mites in relation to the number of bees in the sample.

### 2.3. Colony Morphotype

The morphotype of the colonies was determined using the Fast Africanized Bee Identification System (FABIS I) [44]. The right forewings of 10 workers of each colony were detached, placed over transparent tape and mounted between two 24 × 50 mm coverslips. Each mount containing 10 forewings was scanned to obtain a digital image, which was used to measure the length of each wing using the Motic Image Plus 2.0 software to obtain the mean forewing length of the colony [42,45].

Honeybee colonies with an average forewing length ≥ 9.095 mm were classified as the European morphotype, colonies with an average length ≤ 8.950 mm were classified as the Africanized morphotype and the colonies with an average forewing length between 8.951 and 9.094 mm were classified as the Intermediate or Hybrid morphotype [44].

### 2.4. Colony Haplotype

To determine the haplotype of the colonies, DNA was extracted from three workers of each colony. Each worker was homogenized in lysis solution (1% CTAB, 50 mM Tris pH 8.0, 10 mM EDTA, 1.1 M NaCl), followed by phenol/chloroform extraction and ethanol precipitation of the DNA [46]. The DNA of each bee was quantified and diluted to a final concentration of 100 ng/µL.

Using the DNA extracted from each honeybee, two PCR-RFLP markers located at the mitochondrial DNA were generated to determine the haplotype of the colonies. Two regions of the mitochondrial DNA, region 2095–3123 of the cytochrome oxidase I (COI) gene and region 13479–14443 of the IsmRNA gene were amplified. The PCR products were digested with Hincll enzyme for the COI gene region and with the EcoRI enzyme for the IsmRNA gene region [47].

The haplotype of the colonies was determined from the genotype generated by the two markers in the DNA of the three workers of each colony. The colonies were classified as the East European haplotype, West European haplotype and African haplotype [47].

### 2.5. Climate Type

To identify the climate type, as well as some climatic elements for each beeyard included in the study, the latitude, longitude and altitude were obtained with a GPS device. The climate type was determined using the climate charts published by the National Institute of Statistics and Geographic Information (INEGI), which are based in Köppen’s climate classification system, modified for the geographical conditions of Mexico [48].

The beeyards were located in regions with a temperate sub-humid climate (C(w)), semi-warm climate (ACw) and warm sub-humid climate (A(w)). The temperate sub-humid climate has a mean annual temperature of 16 °C and a mean annual precipitation of 1300 mm, the semi-warm climate has a mean annual temperature of 21 °C and a mean annual precipitation of 1100 mm, and the warm sub-humid climate has a mean annual temperature of 24 °C and a mean annual precipitation of 800 mm.

Data on the mean annual temperature, mean annual precipitation, winter precipitation and Lang index were obtained for each beeyard from the databases of INEGI [49]. The Lang index is calculated by dividing the total annual precipitation by the mean annual temperature and it is a method to measure the humidity of a region; a high value on this index means high humidity, while a low value on the index means low humidity.

### 2.6. Effect of the Morphotype, Haplotype and Climate Type on the Prevalence of Varroa Destructor

To determine the effect of the honeybee morphotype, the honeybee haplotype and the climate type on the prevalence of Varroa, a homogeneity chi-square test was used to determine if the frequency of infested and non-infested colonies with Varroa was distributed homogeneously between morphotypes, haplotypes and climate types.

### 2.7. Effect of the Morphotype, Haplotype and Climate Type on the Infestation Levels of Varroa Destructor

A Shapiro–Wilk test was used to test if the distribution of the data from the colonies infested by Varroa fitted a normal distribution; the data were transformed using the Box and Cox method to provide a normal distribution to fulfill the assumptions of the analysis of variance.

To determine if the morphotype and haplotype of honeybees, as well as the climate type, have an effect on the infestation levels of *Varroa destructor*, data from the infested colonies were analyzed with an analysis of variance using a general linear model that included the effect of the colony morphotype, the colony haplotype, the climate type and the interactions between these variables.

### 2.8. Effect of Climatic Elements on the Infestation Levels of Varroa Destructor

The Varroa mean infestation level of each beeyard was estimated, and an analysis was performed to estimate the Pearson correlation coefficient between the Varroa mean infestation level of the beeyard and the mean annual temperature, the mean annual precipitation, the winter precipitation and the Lang index.

## 3. Results

### 3.1. Estimation of Prevalence and Infestation Levels of Varroa Destructor

Of the 1134 colonies included in the study, 910 were infested with Varroa and 224 were not infested; the estimated prevalence of Varroa in the population was 0.80. The average level (±SE) of infestation in the colonies was 3.79 ± 0.14%, with a minimum of 0.0% and a maximum of 40.46%.

### 3.2. Colony Morphotype

**Figure 3:**
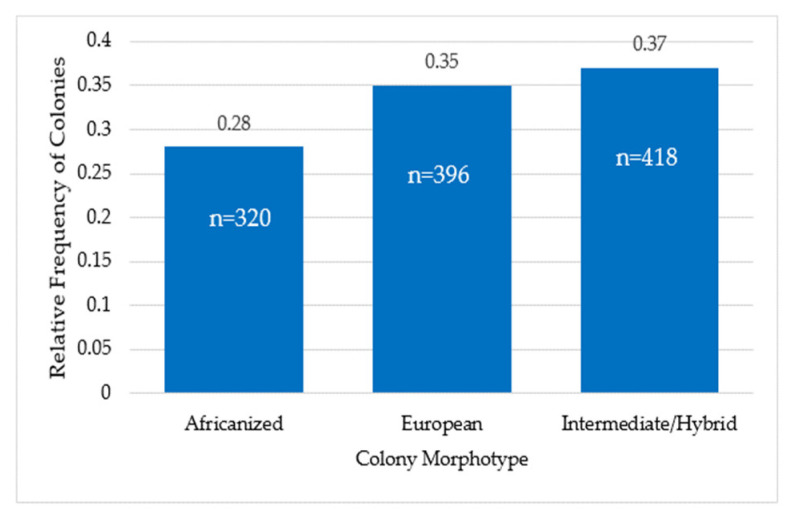
Relative frequency of honeybee colonies with Africanized, European and Intermediate or Hybrid morphotype. Figures inside the bars correspond to the number of colonies.

The relative frequency of colonies with the Africanized morphotype was 0.28 (n = 320), of colonies with the European morphotype was 0.35 (n = 396) and the frequency of colonies with the Intermediate or Hybrid morphotype was 0.37 (n = 418) (Figure 3).

### 3.3. Colony Haplotype

**Figure 4:**
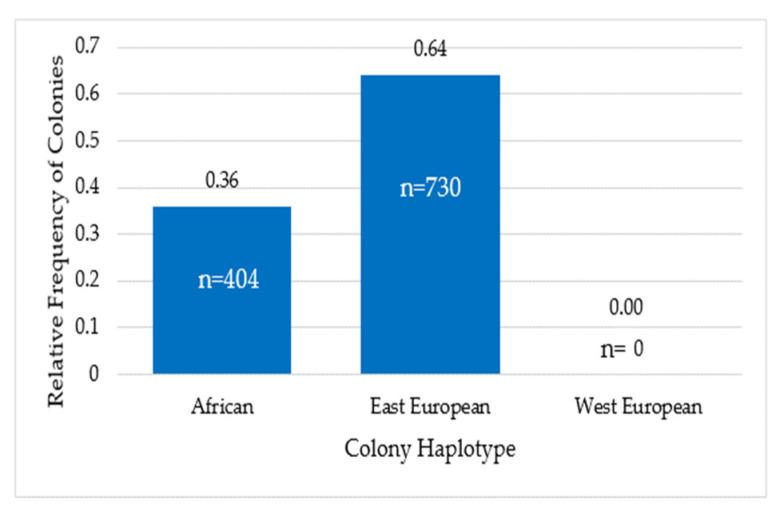
Relative frequency of honeybee colonies with African, East European and West European haplotype. Figures inside the bars correspond to the number of colonies.

The relative frequency of colonies with the African haplotype was 0.36 (n = 404) and the relative frequency of colonies with the East European haplotype was 0.64 (n = 730). Colonies with the West European haplotype were not found in the population (Figure 4).

### 3.4. Climate Type

**Figure 5:**
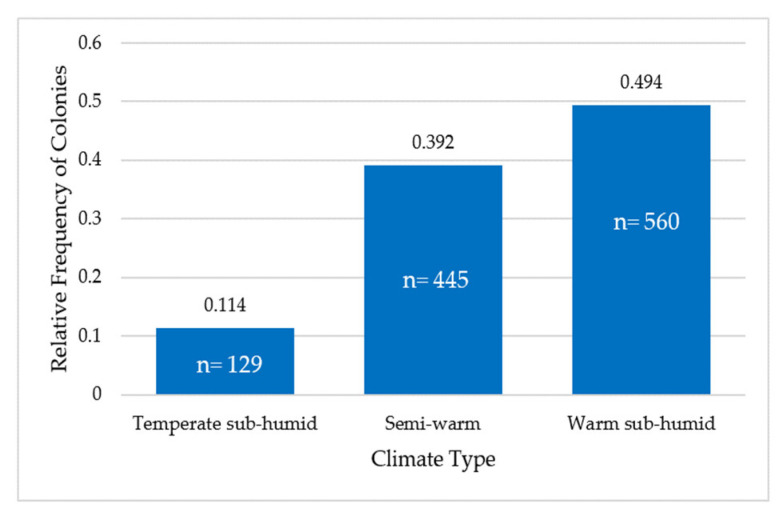
Relative frequency of colonies located in the temperate sub-humid, semi-warm and warm sub-humid climates. Figures inside the bars correspond to the number of colonies.

The relative frequency of colonies located in the temperate sub-humid climate was 0.114 (n = 129), in the semi-warm climate was 0.392 (n = 445) and in the warm sub-humid climate was 0.494 (n = 560) (Figure 5).

### 3.5. Effect of the Morphotype, Haplotype and Climate Type on the Prevalence of Varroa Destructor

The distribution of infested and non-infested colonies was homogeneous in the three honeybee morphotypes (*Xi*^2^ = 0.54; n = 1134; *p* > 0.05) (Table 1). The estimated prevalence of Varroa for honeybee colonies with the Africanized morphotype was 0.82, for colonies with the European morphotype was 0.80 and for colonies with the Intermediate or Hybrid morphotype was 0.79.

The distribution of infested and non-infested colonies was not homogeneous in the two honeybee haplotypes (*Xi*^2^ = 4.72; n = 1134; *p* < 0.05) (Table 2). The estimated prevalence of Varroa for honeybee colonies with the African haplotype was 0.84 and for colonies with the European haplotype was 0.78.

The distribution of infested and non-infested colonies was not homogeneous in the three climate types (*Xi*^2^ = 15.03; n = 1134; *p* < 0.01) (Table 3). The estimated prevalence of Varroa for colonies in the temperate sub-humid climate was 0.93, in the semi-warm climate was 0.79 and for colonies in the warm sub-humid climate was 0.78.

### 3.6. Effect of the Morphotype, Haplotype and Climate Type on the Infestation Levels of Varroa Destructor

No effect of the honeybee morphotype on the Varroa infestation levels was found. There were no differences in the infestation level between the three honeybee morphotypes (F = 0.03; df = 2, 892; *p* > 0.05) (Table 4). The mean infestation level (±SE) of the colonies with the Africanized morphotype was 5.56 ± 0.49%, and that of the colonies with the European morphotype was 5.43 ± 0.32%; the infestation level of the colonies with the Intermediate or Hybrid morphotype was 5.50 ± 0.32%.

No effect of the honeybee haplotype on the Varroa infestation levels was found. There were no differences in the infestation level between the two honeybee haplotypes (F = 0.56; df = 1, 892; *p* > 0.05) (Table 4). The mean infestation level (±SE) of the colonies with the African haplotype was 5.33 ± 0.32% and the infestation level of the colonies with the East European haplotype was 5.67 ± 0.30%.

No effect of the interaction between the honeybee morphotype and haplotype on the infestation levels of Varroa was found (F = 2.52; gl = 2, 892; *p* > 0.05) (Table 4).

An effect of the climate type on the Varroa infestation levels was found. There were differences in the colony infestation level between the three climate types (F = 16.39; gl = 2, 892; *p* < 0.01) (Table 4). The mean infestation level in the temperate sub-humid climate was significantly higher than in the semi-warm climate and the warm sub-humid climate (*p* < 0.05), and the mean infestation level in the semi-warm climate was higher than in the warm sub-humid climate (*p* < 0.05).

**Figure 6:**
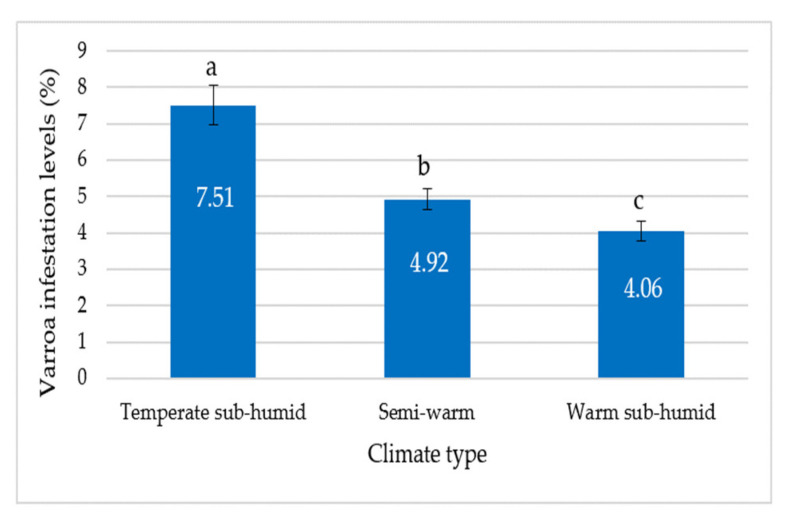
Varroa mean infestation levels (±SE) in the temperate sub-humid, semi-warm and warm sub-humid climates. Figures inside the bars correspond to Varroa mean infestation levels. Different letters indicate differences of Varroa mean infestation levels based on an analysis of variance and a Tukey test (*p* < 0.05).

The mean infestation level (±SE) of the colonies in the temperate sub-humid climate was 7.51 ± 0.54%, in the semi-warm climate was 4.92 ± 0.29% and in the warm sub-humid climate was 4.06 ± 0.27 (Figure 6).

No effects on the infestation levels of Varroa were found for the interactions between morphotype and climate type (F = 2.23; df = 4, 892; *p* > 0.05), between haplotype and climate type (F = 0.62; df = 2, 892; *p* > 0.05), and between morphotype, haplotype and climate type (F = 0.37; df = 4, 892; *p* > 0.05) (Table 4).

### 3.7. Effect of Climatic Elements on the Infestation Levels of Varroa Destructor

**Figure 7:**
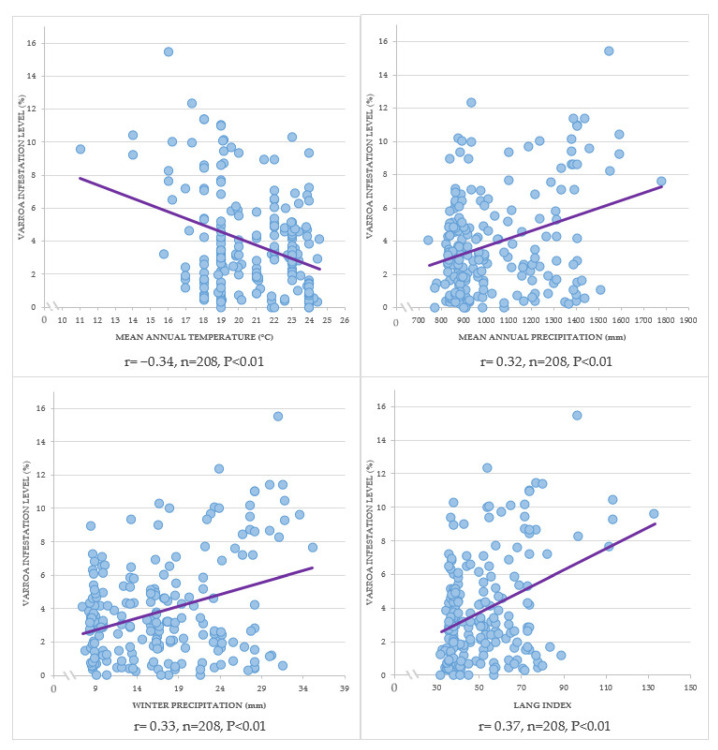
Correlations between the Varroa mean infestation level of the beeyard and climatic elements.

A negative correlation was found between the Varroa mean infestation level of the beeyard with the mean annual temperature (r = −0.34; n = 208; *p* < 0.01). Positive correlations of the Varroa mean infestation level of the beeyard with the mean annual precipitation (r = 0.32; n = 208; *p* < 0.01), with the winter precipitation (r = 0.33; n = 208; *p* < 0.01) and with the Lang index (r = 0.37; n = 208; *p* < 0.01) were found (Figure 7).

## 4. Discussion

The results of this study indicate that honeybee morphotype had no effect on the prevalence and infestation levels of Varroa; that honeybee haplotype had an effect on the prevalence but not on the infestation levels of Varroa, and that climate type had an effect on the prevalence and infestation levels of the mite in the colonies. The results also indicate that the interactions between the honeybee morphotype, honeybee haplotype and climate type had no effect on the prevalence and infestation levels of Varroa.

The prevalence of Varroa did not differ between the three honeybee morphotypes. This result differs from what was reported by two studies also conducted in Mexico, where the prevalence of Varroa was higher in colonies with the European morphotype [40,41]. The results of our study suggest that colonies with the Africanized, European and Hybrid morphotypes have the same probability of being infested and are equally susceptible to the mite.

No differences were found in the infestation levels of the three honeybee morphotypes. A study conducted in Brazil also found that there were no differences in the Varroa infestation levels between Africanized and European honeybees [38]; however our results are different from those of other studies conducted in Brazil and Mexico, where colonies with the Africanized morphotype had lower Varroa infestation levels than colonies with the European and Hybrid morphotypes [34,35,37]. These studies were conducted under experimental conditions and with a small number of colonies (6 to 58); our study was conducted in an open population with a large number of colonies (1134).

The results of this study differ from what was reported by Ramos-Cuellar et al. (2022) [41], who found differences in the infestation levels of the Africanized and European morphotypes in the two climate types included in their study. However, the results partially agree with Medina et al. (2014) [40], who found that there were no differences between the Africanized and European morphotype in one of the three climate types included in their study.

Ramos-Cuellar et al. (2022) [41] and Medina et al. (2014) [40] used the same method that we used in our study to determine the morphotype of the colonies [44]; however, in their studies, only colonies with the European and Africanized morphotypes were included while colonies classified with the Intermediate or Hybrid morphotype were excluded, which is different from our study, in which all three morphotypes were included.

The studies of Ramos-Cuellar et al. (2022) [41] and Medina et al. (2014) [40], were conducted in open populations with 365 colonies and 300 colonies, respectively. This study was conducted in an open population with 1134 colonies, a larger number of colonies than in the two other studies. The sample size influences the capacity of a study to detect differences between the experimental groups. It is possible that the larger number of colonies in our study allowed us to obtain better estimators for the differences between the honeybee morphotypes.

The results of our study suggest that the population growth of Varroa in the colonies is the same for honeybees with the Africanized, European and Intermediate or Hybrid morphotype, and that in a large population of colonies the honeybee morphotype has no effect on the prevalence and infestation levels of *Varroa destructor.*

Some studies suggest that Africanized honeybees are more tolerant to Varroa than European honeybees [34,35,37,50,51]. The results of this study show that in a large population there were no differences in the prevalence and infestation levels of Varroa between Africanized, European and Hybrid honeybees, this suggests that the tolerance to the mite is not linked to a particular morphotype or genetic group. Tolerance to Varroa depends on the expression of mechanisms of resistance to the mite by the honeybees [52,53,54,55,56]; the results of our study suggest that there are no differences in the expression of the mechanisms of resistance in the three morphotypes or genetic groups and, therefore, there are no differences in their tolerance of Varroa.

The prevalence of Varroa was higher in the colonies with the African haplotype; this result is different from the result of another study in which colonies with the European haplotype had a higher prevalence [40]. The results of the two studies indicate that the honeybee haplotype has an effect on the prevalence of Varroa; however, the direction of the effect was different in the two studies. More studies are needed to understand the effect of the honeybee haplotype on the prevalence of Varroa.

The infestation levels of Varroa were no different between the African and East European haplotypes; this result is similar to the result of Medina et al. (2014) [40] and suggests that the honeybee haplotype has no effect on the infestation levels of Varroa.

The climate type had an effect on the prevalence of Varroa. This result is different from the results of other studies [41,57], but is similar to the result of Medina et al. (2014) [40], who also found an effect of the climate type on the prevalence of Varroa.

In this study, the proportion of infested colonies was higher in the temperate sub-humid climate (16 °C; 1300 mm) than in the semi-warm climate (21 °C; 1100 mm) and the warm sub-humid climate (24 °C; 800 mm). Prevalence was higher in the climates with the lower mean annual temperature and the higher mean annual precipitation of the three climate types included in the study. Medina et al. (2014) [40] found that prevalence was higher in the temperate sub-humid climate (21 °C; 1100 mm) and the subtropical climate (22 °C; 850 mm) than in the temperate dry climate (15 °C; 470mm).

This study and the study of Medina et al. (2014) [40] were conducted in different geographic regions of Mexico; in both studies, the temperate sub-humid climate had the highest prevalence of Varroa. This suggests that the conditions of this climate type are more adequate for Varroa to infest colonies in comparison with the other climate types included in the two studies.

The climate type had an effect on the infestation levels of Varroa. This result is different from what was reported in other studies [41,57], but is similar to the results of other studies that also found an effect of the climate type on the infestation levels of Varroa in the colonies [37,40].

In this study, the infestation level of Varroa was significantly higher in the temperate sub-humid climate (16 °C; 1300 mm) than in the semi-warm climate (21 °C; 1100 mm) and the infestation levels in these two climates were significantly higher than the infestation level found in the warm sub-humid climate (24 °C; 800 mm). The infestation level was higher in the climate with the lower mean annual temperature and the higher mean annual precipitation of the three climates included in the study. Moretto et al. (1991) [37] found that the infestation levels were higher in temperate and cold climates in comparison with warm climates. Something similar occurs in this study: the level of infestation was higher in the temperate climate than in the semi-warm and warm climates. In our study, a gradient in the infestation levels can be observed: the infestation levels increased as the mean annual temperature decreased and as the mean annual precipitation increased in the three climates included in the study.

The Varroa mean infestation level of the beeyards correlates with the climatic elements analyzed in the study; this differs from another study, where no correlations between the infestation levels of Varroa and the climatic elements were found [57], but partially agrees with the results of Harris et al. (2003) [58], who found that the Varroa population growth in the colonies was correlated with some climatic elements.

In this study, a negative correlation between the Varroa mean infestation level of the beeyard and the mean annual temperature was found; in the study of Harris et al. (2003) [58] a negative correlation between the Varroa population growth in the colonies and the average daily maximum temperature was found. In our study, a positive correlation between the Varroa mean infestation level of the beeyard and the Lang index, which is a measure of humidity in a region, was found; however, in the study of Harris et al. (2003) [58], a positive correlation between the Varroa population growth and the average daily relative humidity was found. However, in this study, positive correlations of the Varroa mean infestation level of the beeyard with the mean annual precipitation and winter precipitation were found, and in the study of Harris et al. (2003) [58], no correlation was found between the Varroa population growth in the colonies and the average daily rainfall.

The correlations found in this study suggest that the temperature and humidity in a region have an effect on the population growth of Varroa in the colony and on the infestation levels that Varroa can reach in the colonies.

## 5. Conclusions

The overall results of the study indicate that the genetic origin of honeybees has no effect on the prevalence and infestation levels of *Varroa destructor*, that climate is an important factor that affects the prevalence and the infestation levels that the mite can reach in the colonies, and that the interaction between the genetic origin of the honeybees and the climate do not affect the prevalence and infestation levels of *Varroa destructor*.

## Figures and Tables

**Table 1:** Number of honeybee colonies with Africanized, European and Intermediate/Hybrid morphotype, infested and non-infested with Varroa.

Colony Morphotype	Number of Varroa-Infested Colonies	Number of Varroa-Free Colonies
Africanized	261 (0.82)	59 (0.18)
European	317 (0.80)	79 (0.20)
Intermediate/Hybrid	332 (0.79)	86 (0.21)

Numbers in parentheses correspond to the relative frequency.

**Table 2:** Number of honeybee colonies with African and European haplotype, infested and non-infested with Varroa.

Colony Haplotype	Number of Varroa-Infested Colonies	Number of Varroa-Free Colonies
African	338 (0.84)	66 (0.16)
European	572 (0.78)	158 (0.22)

Numbers in parentheses correspond to the relative frequency.

**Table 3:** Number of honeybee colonies located in temperate sub-humid, semi-warm and warm sub-humid climates, infested and non-infested with Varroa.

Climate Type	Number of Varroa-Infested Colonies	Number of Varroa-Free Colonies
Temperate Sub-humid	120 (0.93)	9 (0.07)
Semi-warm	351 (0.79)	94 (0.21)
Warm sub-humid	439 (0.78)	121 (0.22)

Numbers in parentheses correspond to the relative frequency.

**Table 4:** Generalized Linear Model (GLM) analysis of Varroa infestation level in honeybee colonies.

Source	Sum of Squares	DF	Mean Square	F	*p*
Model	1823.228	17	107.249	4.6621	<0.0001
Morphotype	1.2185	2	0.6092	0.0097	0.9904
Haplotype	13.0152	1	13.0152	0.0363	0.8490
Climate Type	754.2876	2	377.1438	15.9460	<0.0001
Morphotype × Haplotype	115.9339	2	57.9669	0.1045	0.9008
Morphotype × Climate Type	205.3501	4	51.3375	1.7150	0.1446
Haplotype × Climate Type	28.4102	2	14.2051	0.8476	0.4288
Morphotype × Haplotype × Climate Type	33.7994	4	8.4498	0.5815	0.6762
Error	20,519.930	892	23.0044		
Total	22,343.158	909			

## Data Availability

The data analyzed in the study can be provided by the corresponding author upon reasonable request.
